# Forthcoming hyperfluorescence display technology: relevant factors to achieve high-performance stable organic light emitting diodes

**DOI:** 10.3389/fchem.2023.1211345

**Published:** 2023-06-12

**Authors:** Yogesh Gawale, Rasheeda Ansari, Kenkera Rayappa Naveen, Jang Hyuk Kwon

**Affiliations:** Organic Optoelectronic Device Lab (OODL), Department of Information Display, Kyung Hee University, Seoul, Republic of Korea

**Keywords:** hyperfluorescence, OLEDs, electroluminescence, FRET, external quantum efficiency

## Abstract

Over the decade, there have been developments in purely organic thermally activated delayed fluorescent (TADF) materials for organic light-emitting diodes (OLEDs). However, achieving narrow full width at half maximum (FWHM) and high external quantum efficiency (EQE) is crucial for real display industries. To overcome these hurdles, hyperfluorescence (HF) technology was proposed for next-generation OLEDs. In this technology, the TADF material was considered a sensitizing host, the so-called TADF sensitized host (TSH), for use of triplet excitons via the reverse intersystem crossing (RISC) pathway. Since most of the TADF materials show bipolar characteristics, electrically generated singlet and triplet exciton energies can be transported to the final fluorescent emitter (FE) through Förster resonance energy transfer (FRET) rather than Dexter energy transfer (DET). This mechanism is possible from the S_1_ state of the TSH to the S_1_ state of the final fluorescent dopant (FD) as a long-range energy transfer. Considering this, some reports are available based on hyperfluorescence OLEDs, but the detailed analysis for highly efficient and stable devices for commercialization was unclear. So herein, we reviewed the relevant factors based on recent advancements to build a highly efficient and stable hyperfluorescence system. The factors include an energy transfer mechanism based on spectral overlapping, TSH requirements, electroluminescence study based on exciplex and polarity system, shielding effect, DET suppression, and FD orientation. Furthermore, the outlook and future positives with new directions were discussed to build high-performance OLEDs.

## 1 Introduction

Organic light-emitting diodes (OLEDs) are colored light sources effectively used in manufacturing the displays of smartphones and television screens and lighting for various applications. The performance of the OLEDs in commercial displays remarkably improved from the first generation to the current generation technologies in the last 2 decades (Hong et al., 2021). Each new technology breaks the barriers to previous technology regarding electroluminescent efficiency, color purity, manufacturing cost, lifetime, etc. The success of OLED displays can be measured by betterment in resolution, wideness of viewing angles, prompt response time, high contrast colors, and the possibility of realizing transparency in OLED displays (Wong and Zysman-Colman, 2017). The most recent winning argument is also the realization of flexible OLED displays shown in SID 2022 and in other exhibitions and international conferences on display technology, such as OLED Korea Conference, OLEDs World Summit, Organic and Printed Electronics Convention, Electronics display Conference, and SPIE Photonics west including advertisement on televisions that can be bent and rolled up like a poster, which opens a new dimension to the design possibilities of displays entirely (Li et al., 2013).

Usually, a key challenge in OLEDs is the weak interaction of lowest-lying singlet (S_1_), and lowest-lying triplet (T_1_) states, which slows down the conversion of T_1_ to S_1_ states of organic materials. To operate the OLED device, the efficient conversion is a crucial factor because, in contrast to the selection rules associated with photo excitation, the process of generating the excitons on the emitter in OLEDs (electrical excitation) leads to a singlet to triplet population ratio of 25:75 due to spin statistics of charge recombination (Wilson et al., 2001) as the consequences, in the case of first-generation fluorescent OLEDs (FL-OLEDs), only 25% of generated excited states will be harvested. So, the internal quantum efficiency (IQE) is limited to only 25% in FL OLEDs (Xu et al., 2021). In order to achieve 100% IQE and utilize the remaining 75% excitons in the triplet state, spin-orbit coupling is promoted with the support of intersystem crossing (ISC) by embedding metal atoms, such as platinum (Pt), iridium (Ir), and gold (Au), in the molecules, resulting in the second-generation phosphorescent materials (Baldo et al., 1998; Reineke et al., 2007). Furthermore, singlet excitons are formed by combining the two triplet excitons through triplet–triplet annihilation (TTA), which results in delayed electroluminescence (Seung Hyun Lee et al., 2023). However, in conventional organic materials, usually at low temperature, the direct radiative decay of triplet excitons shows phosphorescence (Uoyama et al., 2012). Therefore, using heavy-metal atoms limits the further market expansion of phosphorescent materials considering the environmental concerns and cost issues (Volz et al., 2015).

To overcome the cost issues, the pioneering work of Adachi et al., in 2012 on thermally activated delayed fluorescence (TADF) (Zhang et al., 2012; Adachi, 2014; Zhang Q. et al., 2014) attracted great attention regarding the utilization of these materials in OLED applications. These are known to be the third-generation OLED technologies. The significant advantages of utilizing TADF materials are low-cost and use of environmentally precarious elements avoiding metal atoms while offering high IQE (Yang et al., 2017). Furthermore, TADF is being explored and examined with great interest in industrial and academic research (Pratik et al., 2022). The uniqueness of TADF molecules is the small energy gap (ΔE_ST_) between the lowest-lying singlet state S_1_ and lowest-lying triplet state T_1_ due to well-separated frontier molecular orbitals (FMOs) (Barman et al., 2020; Lee et al., 2020; Yin et al., 2020). When the T_1_ excitons’ lifetime is long enough, the process of spin-forbidden reverse intersystem crossing (RISC) becomes thermally activated and facilitates the up-conversion of triplet excitons into the S_1_ state, through which they relax radiatively to the ground state, and leads to a theoretical IQE of 100% (Yang et al., 2017). In OLEDs, the emitted light is generated by the recombining electrons and holes in the organic layer. However, only a fraction of the energy applied to the OLED is converted into light, with the rest being lost as heat. This limits the efficiency of OLEDs and makes them less bright than other types of lighting devices. On the other hand, TADF OLEDs showed broad emission spectra (Zhang et al., 2012; Nakanotani et al., 2014; Chen et al., 2017; Guo et al., 2017; Hosokai et al., 2017; Chen et al., 2018; Penfold et al., 2018; Shin et al., 2019; Wada et al., 2020; Cho et al., 2022; Rayappa Naveen et al., 2022), which jeopardizes the requirements for BT2020 and National Television Standards Committee (NTSC) (Sugawara et al., 2014; Matsuo and Sakaida, 2016; Wang M. et al., 2019).

**FIGURE 1:**
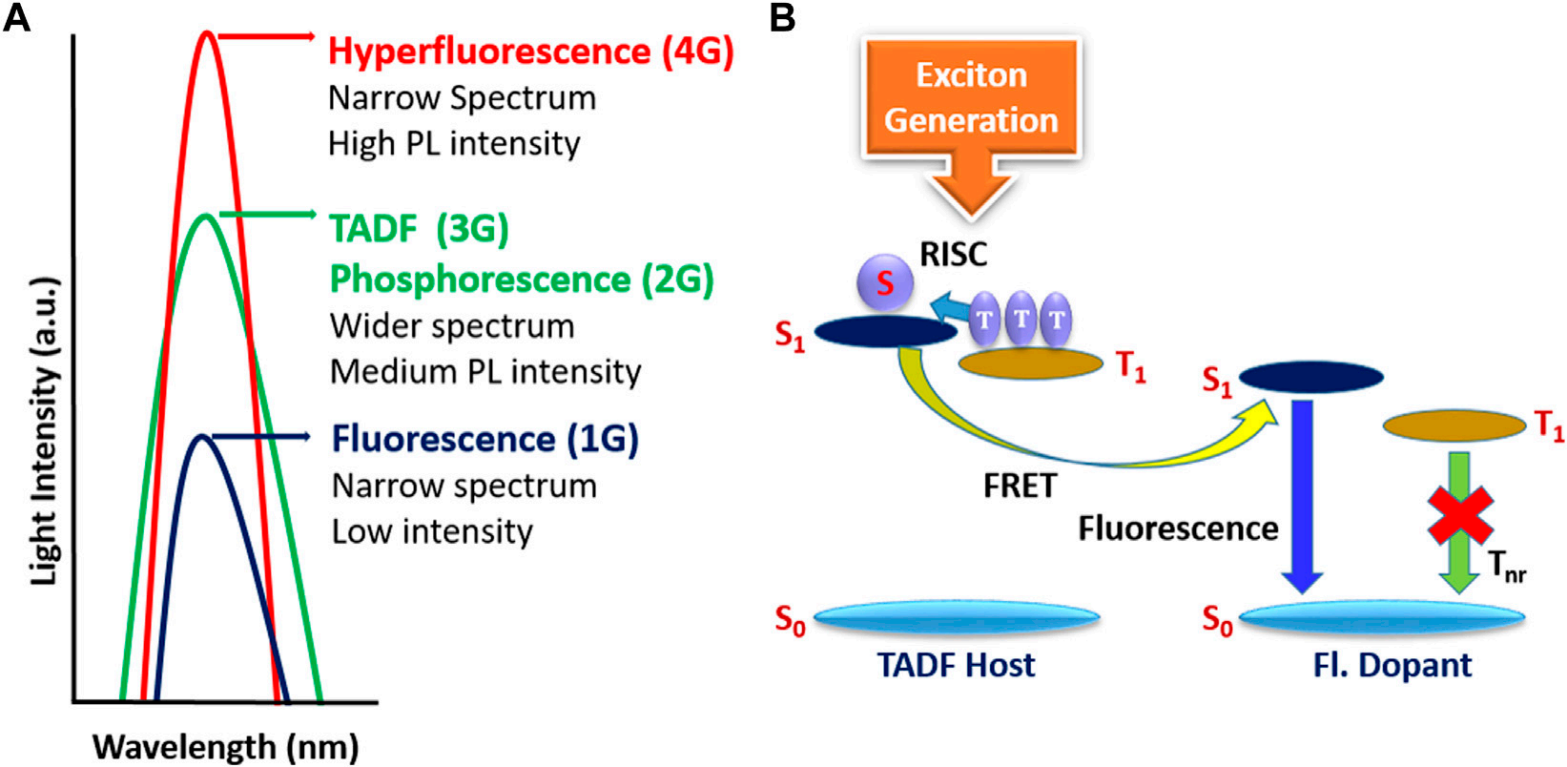
**(A)** Spectral behavior of various generations. **(B)** Basic hyperfluorescence mechanism in OLEDs.

Hatakeyama et al. developed multiresonant TADF (MR-TADF) materials, which is a focus of keen research these days (Hatakeyama et al., 2016; Madayanad Suresh et al., 2020; Naveen et al., 2023). This subclass of TADF materials, so-called MR-TADF, shows significant advantages, such as narrow full width at half maximum (FWHM), high photoluminescence quantum yield (PLQY), and color purity, which are highly desirable for real display applications. However, the EQE is limited to 20–30% in most of the MR-TADF molecules (Naveen et al., 2022b; Konidena and Naveen, 2022), except a few reporting an efficiency of more than 30% (Kondo et al., 2019; Cheng et al., 2022; Wang et al., 2023). On the other hand, the fourth-generation hyperfluorescence (HF) technology was developed (Nakanotani et al., 2014; Furukawa et al., 2015), which combines TADF assistant host (TSH) and final fluorescent narrowband dopant (FD) (Nakanotani et al., 2014). This technology can allow obtaining high EQE and narrowband emission with high color purity, resulting in wider color gamut displays (Braveenth et al., 2021; Jiang et al., 2022; Fan et al., 2023; Hu et al., 2023). The detailed spectral behaviors with all fourth-generation HF technologies and the detailed HF device mechanism are illustrated in Figure 1. HF technology has been used in commercial OLED displays and lighting products, and further research is ongoing to improve its performance and efficiency (Kim E. et al., 2022; Sun et al., 2022).

Even though there are some reports on HF-OLEDs, there needs to be a clear understanding of the related factors. So herein, we reviewed the essential requirements such as spectral overlapping, energy transfer mechanisms, TSH requirements, electroluminescence study based on the exciplex and polarity system, shielding effect, DET suppression, and FD orientation by considering the molecular design concepts and device performances for future consideration of HF-OLEDs.

## 2 Factors to achieve highly efficient and stable HF-OLEDs

### 2.1 Spectral overlapping for energy transfer processes

Spectral overlapping refers to the degree of overlap between the absorption spectra of the fluorescent emitter and the emission spectra of the assistant host (charge transport materials) (Bennett, 2004; Duan et al., 2022). When the emission spectrum of the assistant host, usually TSH, overlaps with the absorption spectrum of the final fluorescent dopant, some of the energy from the emitter is absorbed, and thus more efficient energy transfer can be anticipated (Nakanotani et al., 2014; Cravcenco et al., 2020). Usually, spectral overlapping is crucial and determines the energy transfer efficiency between the TADF assistant host and the narrowband final dopant in achieving high-performance HF OLEDs (Nakanotani et al., 2014; Furukawa et al., 2015). Therefore, the tuning of spectral overlapping in OLEDs is critical in achieving high-quality and efficient emission, and this requires a combination of advanced material design, device optimization, and simulation techniques. Förster resonance energy transfer (FRET) is a non-radiative energy transfer process between two neighboring fluorescent molecules, in which one fluorescent molecule absorbs light and transfers its energy to another molecule via FRET. The efficiency of FRET can be measured with respect to the Förster radius distance (R_F_), which represents the molecular separation at which the energy transfer is 50% efficient (Dacres et al., 2010). R_F_ can be expressed by the following equation:

$$R_{F}^{6}=\frac{9000\left(\ln10\right)\Phi_{D}\kappa^{2}}{N_{A}128\pi^{5}n^{4}}\int_{0}^{\infty }F_{D}\left(\lambda \right)\varepsilon_{A}\left(\lambda \right)\lambda^{4}d\lambda$$

where R_F_ is the distance at which the energy transfer is taken as 50% (Förster radius distance), N_A_ is Avogadro’s number, *n* is the refractive index of the medium, *k* ^2^ is an orientation factor, Φ is the quantum yield of TSH, and F_D_ (λ) and ε_A_(λ) are the normalized PL spectra of TSH and molar absorption coefficient of the dopant, respectively. Hence, a large R_F_ value is required to achieve higher FRET efficiency (Naveen et al., 2022a). High R_F_ can be attained with large spectral overlapping due to the increase in Förster radius distance and intermolecular distances between TSH and FD.

**FIGURE 2:**
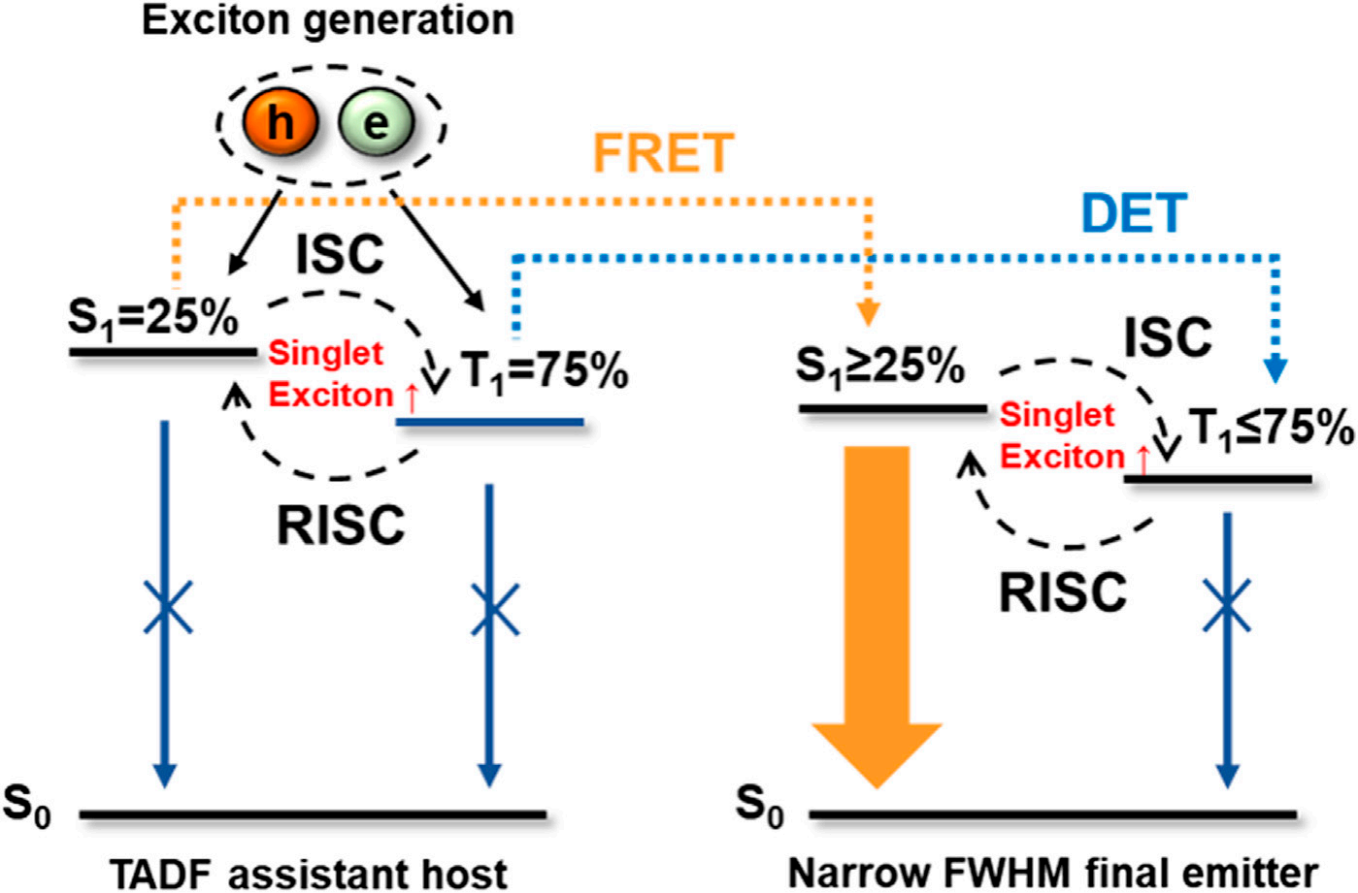
Schematic representation of FRET and DET mechanisms in the hyperfluorescence system.

**FIGURE 3:**
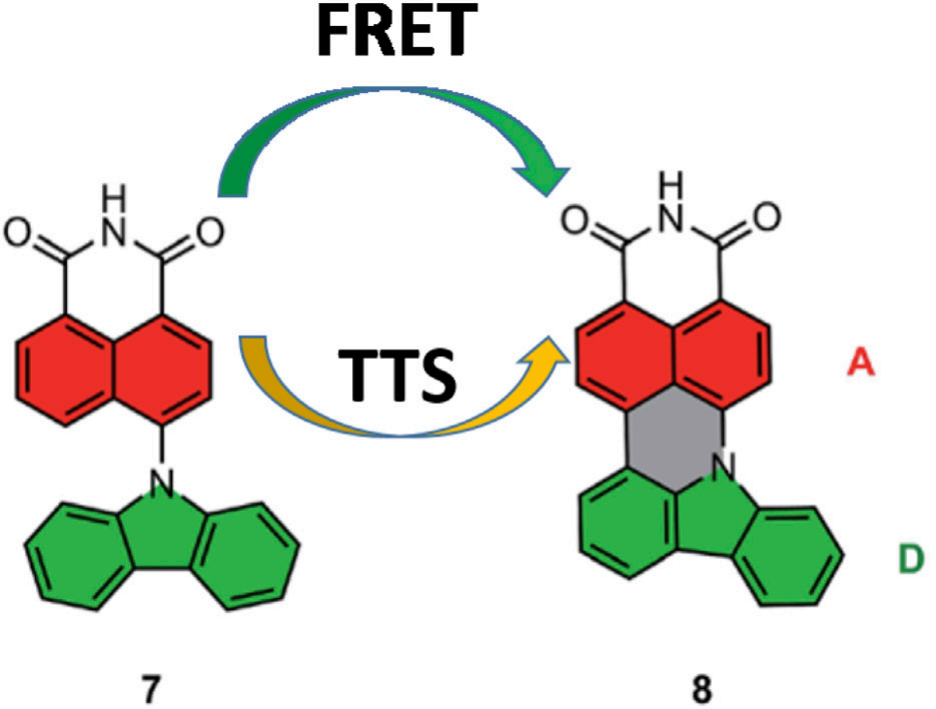
Molecular design toward double harvesting of the triplet via FRET and TTS.

In 2014, Adachi et al. utilized the FRET approach to propose efficient HF OLEDs with high color purity and enhanced stability (Nakanotani et al., 2014). In this approach, as illustrated in Figure 2, the narrow FWHM final emitter and TADF assistant host were embedded in a host matrix so that FRET can occur from the S_1_ of a TADF assistant host to the S_1_ of a final emitter (Kim et al., 2015; Song et al., 2015; Ahn et al., 2018; Zhang et al., 2018; Adachi et al., 2019; Byeon et al., 2019). In addition, the narrow emission spectra of dopants and the high radiative decay rate of sensitizers are responsible for fast and complete FRET, which increases the color purity and stability of the OLED (Kim et al., 2015; Liu et al., 2015). Furthermore, Lindner et al., reported a rational approach to simultaneously pursue FRET and triplet-to-singlet (TTS) transition as complementary mechanisms for effective exciton transfer, realizing an EQE up to 27% and FWHM of 40 nm (Bartkowski et al., 2022). Figure 3 shows the representative structure of the TADF sensitizer (7) and fluorescent dopant (8), where the D moiety in compound 7 is twisted to the A moiety. The S_1_ arises from intramolecular DA charge transfer (ICT), whereas, in compound 8, the additional linkage between the electron-accepting and -donating moieties has a strong effect on the electronic structures of the S_1,_ which is a π–π*-type state that is delocalized over both the D and A moieties. Hence, it does not show a substantial ICT characteristic. In order to achieve high efficiency, the concentration ratio of the host, TADF, and fluorescence dopant is to be optimized so that it maximizes the FRET rate (Yi et al., 2020). This is an effective strategy to increase the color purity and the stability of highly efficient HF-OLEDs.

Furthermore, Penfold et al., demonstrated the model of the HF process between a 2,5,8,11-tetra-tert-butylperylene (TBpe), narrow band blue fluorescent emitter and carbene–metal–amide (CMA) molecule with the Au bridging metal (Au-Cz) through quantum chemistry and quantum dynamics simulations (Giret et al., 2021). In this study, the high FRET rate encouraged rapid triplet harvesting which is strongly dependent on the difference in energy between the S_1_ states of the acceptor and donor molecules. The FRET rate of 10^10^ s^-1^, illustrated by quantum dynamics simulations, indicates that it occurs on the picosecond timescale comparable with the ISC crossing rate of Au-Cz. These quantum dynamics simulations could enhance the understanding of HF-based emitters.

Hence, in order to satisfy the FRET rate in the HF system, the TADF sensitizer and emitters should be chosen with respect to their distance, which should be relatively close; the short excited state lifetime of the TADF sensitizer; the high absorption coefficient of the final emitter; and overlapping of the absorption and emission spectra of a final emitter and TADF sensitizers.

### 2.2 TADF host requirements

TADF is a mechanism that allows for efficient harvesting of both singlet and triplet excitons in OLEDs. Conventional fluorescence molecules, where triplet excitons would non-radiatively decay from triplet to the ground state, would not contribute to light emission and cannot be used as TADF sensitizers because of the large ΔE_ST_. When TADF materials are used as a TSH, the triplet excitons on the TSH are up-converted to singlet excitons by the RISC process (Yoon et al., 2021). Hence, it is crucial to utilize triplet excitons for TSH efficiently. The prerequisite for the materials to be used as TADF sensitizers is their smaller ΔE_ST_ to induce RISC and their participation in effective FRET, which can relieve exciton quenching and improve the device performance (Nakanotani et al., 2014). Therefore, the TADF sensitizer and final emitter should be carefully chosen for effective FRET. Usually, TSH can convert triplet excitons into singlet excitons through an RISC process, which then allows for the transfer of these excitons to the final fluorescent emitters (Nakanotani et al., 2014). This mechanism has been shown to enable high external quantum efficiencies in OLEDs (Tanaka et al., 2012).

In 2014, Adachi et al. introduced TADF materials as sensitizers in HF devices (Nakanotani et al., 2014). The series of molecules with small ΔE_ST_ such as 10-phenyl-10H,10′H-spiro [acridine-9,9′-anthracen]-10′-one (ACRSA), 3-(9,9-dimethylacridin-10-(9H)-yl)-9H-xanthen-9-one (ACRXTN), 2-phenoxazine-4,6-diphenyl-1,3,5-triazine (PXZ-TRZ), and 2,4,6-tri (4-(10Hphenoxazin-10H-yl)phenyl)-1,3,5-triazine (Tri-PXZ-TRZ) as a TSH and the corresponding fluorescent molecules 2,5,8,11-tetra-tert-butylperylene (TBpe), 9,10-Bis [N,N-di-(p-tolyl)-amino]anthracene (TTPA), 2,8-ditert-butyl-5,11-bis(4-tert-butylphenyl)-6,12-diphenyltetracene (TBRb), and tetraphenyldibenzoperiflanthene (DBP) were selected as final emitters in such way that the emission spectra of all TSH and the absorption spectra of the final emitters get overlapped. Blue, green, yellow, and red HF devices, via an energy transfer cascade, were prepared using these materials and realized the EQE_max_ of 13.4–18%. This strategy is most beneficial to develop HF devices. Since then, several efforts have been reported to increase the fluorescent intensity up to four times that of the conventional fluorescent emitters, solving the wide spectrum problem of TADF materials. For example, Cui et al. (2020) reported the sky-blue TADF material 5Cz-TRZ consisting of multiple carbazole donors and one triazine acceptor. The RISC rate of 5Cz-TRZ is 1.5×10^7^ s^-1^, which is nearly two orders of magnitude higher than the typical values for TADF emitters. This higher RISC rate is due to the formation of charge-resonance-type hybrid triplet states by multiple carbazole donors. This resulted in the large spin-orbit coupling, small singlet–triplet energy splitting, and dense triplet-state manifold. The HF device using 5Cz-TRZ as a sensitizer reaches the EQE_max_ value of over 22%. This design strategy paves the way to achieve efficient and stable TADF and HF OLEDs using TADF sensitizers with higher *k*_RISC_ rates.

**FIGURE 4:**
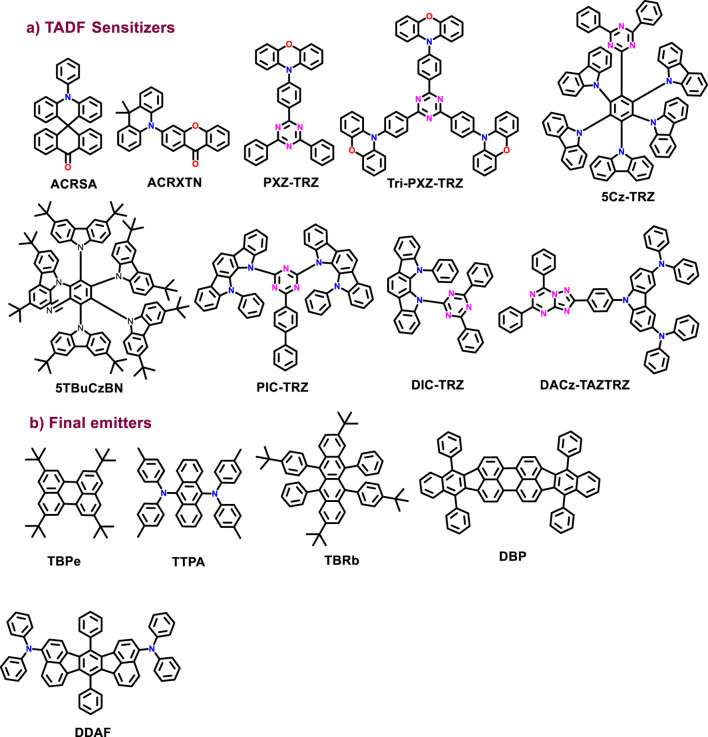
Molecular structures discussed in TADF host requirements without MR-TADF nature.

The performances of TADF-sensitized HF device are shown in Table 1. Qiu et al. demonstrated a general technique to improve the efficiencies of fluorescent OLEDs (FOLEDs) (Zhang D. et al., 2014). The new approach to realizing TADF uses sensitizing hosts with small singlet–triplet energy. This approach differs from traditional methods of using molecules with TADF as emitters in FOLEDs. The host’s singlet excitons, directly generated and up-converted ones from the electrically generated triplets, are transferred to the final emitters’ singlets via FRET and then decay radiatively to give out emitter fluorescence. This work demonstrated an approach that achieves EQEs of up to 23.4% and low-efficiency roll-offs. Jingsong You et al. used triazolotriazine (TAZTRZ) as an acceptor, which has strong electron-withdrawing power to combine with the 3,6-(diphenylamine)carbazole (DACz) donor forming an efficient yellow HF device (Su et al., 2021). The electron-withdrawing ability of TAZTRZ leads to the well-separated distribution of their FMOs and thus reduces the ΔE_ST_, giving rise to fast RISC. The HF device based on DACz-TAZTRZ achieved an EQE_max_ of 23.7%, considered an excellent performance for yellow FOLEDs. The chemical structures are illustrated in Figure 4.

**TABLE 1:** Summarized device performances of the TADF-sensitized HF OLEDs.

TADF sensitizer	Final emitter	Host	EQE_max_ (%)	Ref
ACRSA	TBPe	DPEPO	13.4	Nakanotani et al. (2014)
ACRXTN	TTPA	mCP	15.8	
PXZ-TRZ	TBRb	mCBP	18	
Tri-PXZ-TRZ	DBP	CBP	17.5	
5Cz-TRZ	TBPe	mCBP	24	Cui et al. (2020)
PIC-TRZ	DDAF	-	4.5	Zhang et al. (2014a)
DIC-TRZ	DDAF	-	11.7	
DACz-TAZTRZ	TBRb	32alCTRZ	23.7	Su et al. (2021)
TBE01	PtON-TBBI	SiCzCz:SiTrzCz2	25.4	Kim et al. (2022a)
TBE02	PtON-TBBI	SiCzCz:SiTrzCz2	25.8	
5TBuCzBN	BN-STO	mCBP	40.1	Hu et al. (2023)
Ir (piq)2acac	BNNO	-	34.4	Fan et al. (2023)

**FIGURE 5:**
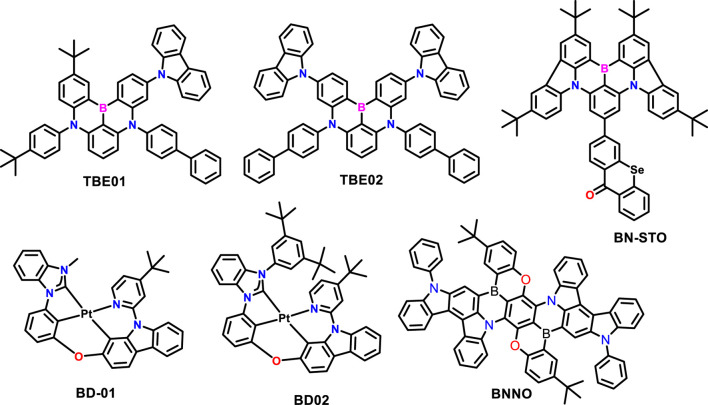
Very recent reported molecular structures based on MR and Pt complexes discussed in TADF host requirements.

Recently, Kim et al. (2022a) developed highly efficient and stable deep-blue OLEDs using phosphor-sensitized TADF (Kim et al.) to overcome the low efficiency of fluorescent OLEDs by utilizing TADF and phosphorescent mechanisms. The new MR-type TADF emitters were developed using a carbazole unit at the periphery of the DABNA core. The carbazole unit at the boron’s para position helped reduce the triplet excitons and trapped charges within the emissive layer. In addition, the carbazole-based derivative emitter helped mitigate the effects of triplet and polaron-related device degradation. Their fast RISC, weak hole trapping, and high molar extinction coefficient made them one of the promising candidates for phosphor-sensitized TADF. Moreover, the bulky peripheral derivatives suppressed the Dexter energy transfer (DET) from nearby molecules, such as the sensitizer and host, to the emitter. In addition, the high absorption coefficient of the emitters enhances the FRET rate from the phosphor sensitizer (PS). The system uses an efficient TADF blue emitter (TBE01 and TBE02) combined with a PS, shown in Figure 5. The emission path is divided into two processes: prompt decay emission arises from the singlet state emitter transferred from the PS and delayed decay emission from excitons up-converted from inevitably populated triplet states in the emitter. The resulting OLED exhibited an operational lifetime of 72.9 h, and the CIE chromaticity coordinates y = 0.165, which is 6.6 times longer than those of devices using conventional TADF emitters. This research provides valuable insights into developing highly efficient and stable blue OLEDs using TADF and phosphorescent mechanisms, which can be used as an alternative to FOLEDs with low EQE.

In the same year, Kim E. et al. (2022) reported that blue phosphorescent OLEDs (PhOLEDs) have superior electroluminescence efficiencies than blue FOLEDs (Sun et al., 2022). Using benzimidazolium carbene as a strong σ-electron donor significantly improves the stability and efficiency of blue PhOLEDs. Introducing bulky 3,5-di-tert-butyl-phenyl into the N-heterocyclic carbene moiety in the Pt (II) complex not only improves the photophysical properties of BD-02 but also makes the BD-02 dopant molecule more stable, which is essential for developing stable and efficient blue PhOLEDs. In addition, the steric hindrance of BD-02 also prevents spectrum broadening from host–guest interactions with the ET host. The introduction of bulky 3,5-di-tert-butyl-phenyl enhances the photochemical stability of the high-lying metal-centered triplet state. It prevents undesirable host–guest interactions, contributing to a longer device lifetime and higher color purity. The blue PhOLED device, using these new materials, exhibited a lifetime of LT_70_ = 1,113 h at L_0_ = 1,000 cd/m^2^, CIE y = 0.197, and EQE_max_ over 20%.

The most recent report by Yang et al. (2017) suggests that employing the peripherally heavy atom decorated strategy successfully achieves high-performance pure green electroluminescence with a maximum EQE of over 40% and reduced efficiency roll-off in corresponding OLEDs (Hu et al., 2023). As shown in Figure 5 (BN-STO), the molecular design involves peripherally decorating multi-resonance TADF emitters with selenoxanthone to achieve a more significant rate of RISC and narrow FWHM by heavy-atom effect. Integrating selenium atoms into the MR-TADF skeleton is more effective in promoting spin-orbit coupling interaction than the peripheral decoration of selenium atoms on the MR-TADF part. The HF technology was used to improve the electroluminescence performance of OLEDs based on the BN-STO emitter by employing 2,3,4,5,6-pentakis-(3,6-di-tert-butyl-9H-carbazol-9-yl)benzonitrile (5TBuCzBN) as the TADF sensitizer exhibited higher EQE of 38.4%. However, the EQE remained at 26.5% at a high luminance level of 1,000 cd m^-2^. Furthermore, Duan et al. reported the development of a narrowband pure-red multi-resonance emitter fused with an indolocarbazole donor, which simultaneously red-shifts and narrows the emission color (Fan et al., 2023). As a result, BNNO shows an emission maximum at 637 nm with a FWHM of merely 32 nm. To ensure efficient energy transfer, the phosphorescent sensitizer Ir (piq)_2_acac was employed in device fabrication, whose emission spectrum significantly overlaps with the absorption spectrum of BNNO. The corresponding OLEDs realize BT.2020 red coordinates for the first time, achieving an EQE_max_ of 34.4% and an ultra-long LT_95_ of over 10,000 h at an initial luminance of 1,000 cd/m^2^.

Therefore, the two critical requirements that should be fulfilled simultaneously for efficient TADF sensitizers are (1) a small energy gap (ΔE_ST_) between the singlet and triplet excited states, which enhances RISC from triplet excited states (T_1_) to singlet excited ones (S_1_) and (2) a reasonable radiative decay rate (*k*_r_), to overcome competitive non-radiative decay pathways and attain a high photoluminescence efficiency.

### 2.3 Electroluminescence phenomenon

a) Polarity

**FIGURE 6:**
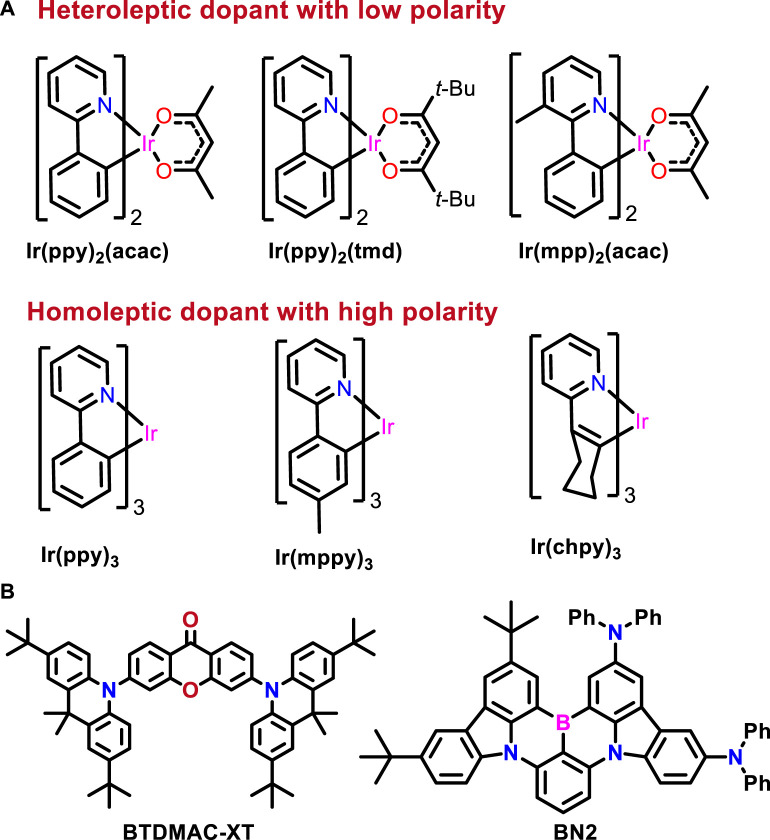
**(A)** Ir(III) complexes. **(B)** Chemical structures discussed based on the polarity concept. **(B)** Exciplex-type device.

When FD emits light by direct recombination, 75% of the exciton is lost along with DET. Therefore, energy loss must be prevented by minimizing FD trapping. On the other hand, trapping has the effect of polarity (μ) of FD in addition to the difference in the energy level between FD and host. Usually, the recombination of charges is an essential process in OLEDs because it influences the driving voltage, efficiency, and lifetime. To understand such a phenomenon, Kim et al. unveiled the recombination mechanism by having six types of dopants using various homoleptic and heteroleptic Ir complexes (three types of low polarity and three types of high polarity) applied to the exciplex host in PSF-OLEDs and compared the degree of trapping (Figure 6) (Lee et al., 2018). The dopant’s dipole moment (μ_0_) rather than the trap depth (ΔE^t^) significantly influences the recombination zone.

As a prerequisite, if FD polarity (μ) increases, the trapping also increases, whereas when the FD polarity (μ) decreases, the trapping and direct recombination also decrease. Among the developed complexes, the homoleptic Ir(III) dyes had large charge trapping due to the large μ_0_, which showed high driving voltage and trap-assisted recombination-dominated emission. However, the heteroleptic Ir(III) complexes showed small μ_0_ and showed less charge trapping irrespective of trap depth, leading to low driving voltage and manifested high EQE values. On the other hand, due to the dipole–charge interaction, the dopant can have a dipole trap, which affects the charge-transporting properties. The trapping characteristics can be described as a Coulombic interaction between the free charge and μ_0_ of the dopant. The trapping strength depends on the polarity of the dopant. The stronger μ_0_ of the dopant is, the more readily it traps an encountered charge. Trapping strength is proportional to the polarity of the dopant derived by Belmont’s equation,

$$S=0.7\left(1+\frac{\sqrt{3}}{4}\right)\frac{q\mu_{0}}{32kT\varepsilon }$$

where S is the capture cross-section and μ_0_ is the static dipole moment. Here, the homoleptic dopants showed 3–4 times higher S values than heteroleptic dopants because of the increased dipole moment values. So, the possibility of trapping or the direct recombination zone can be increased. Even though the discussion is based on Ir complexes, this concept can be vital for designing efficient and stable HF-OLEDs where the recombination sites play an important role. Furthermore, this concept can also allow for building HF-OLEDs with a long lifetime. Very recently, Zhao et al. unveiled a new concept that contains low polarity TADF sensitizer with high steric hindrance and free concentration-quenching for an efficient green HF system (Liu et al., 2023). The developed TADF sensitizer BTDMAC-XT works very well for MR-TADF-type materials. The BTDMAC-XT contains a xanthenone (XT) acceptor and two *tert-butyl* substituted 9,9-dimethyl-9,10-dihydroacridine (BTDMAC) donors. The *tert-butyl* groups can increase the steric hindrance and weaken the molecular polarity. Subsequently, the low polarity material can be attained. Notably, the BTDMAC-XT emitter showed strong delayed fluorescence with a fast RISC process and high PLQY in a neat film. Furthermore, fabricated OLEDs based on the MR-TADF molecule (BN2), with the help of low-polarity host and sensitizers, resulted in a small carrier injection barrier and full exciton utilization. To improve the color quality, the HF-OLEDs are fabricated with low-polar sensitizers for the BN2 molecule and achieved a record high EQEmax of 34.4% for green color with tremendous power efficiency of 166.3 lm W^−1^ and a long operational lifetime (LT_50_ = 40,309 h) at an initial luminance of 100 cd m^−2^. This work can guide future HF-OLEDs as it clearly states that low-polarity-based materials can have great device performances.

**FIGURE 7:**
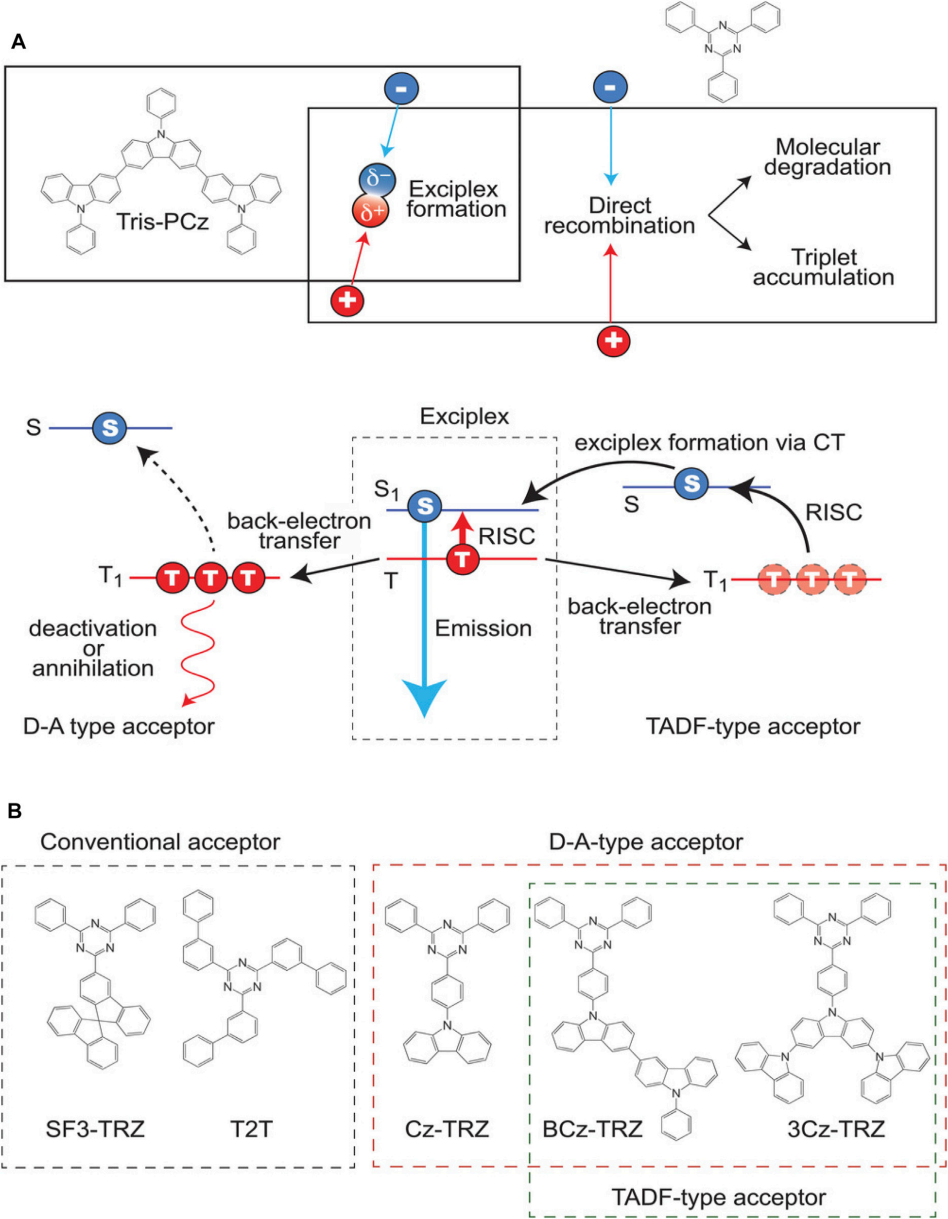
**(A)** Proposed mechanism to improve device stability of the exciplex-based OLED. **(B)** Chemical structure molecules used in this study. *Reproduced with permission from (Nguyen et al., 2020) Copyright ©WILEY-VCH Verlag GmbH and amp; Co. KGaA, Weinheim.*

Usually, when the exciton density inside the emissive layer increases, the device lifetime decreases. Nevertheless, when applying a host with exciplex characteristics, the transfer of holes/electrons to the dopant is minimized compared to normal hosts (Lee K. H. et al., 2023) so that the carrier or triplet accumulation can deteriorate, which helps increase the operational device lifetime (Wang Q. et al., 2019). On the other hand, since all the carriers travel on the host side, the final fluorescent dopant (FD) trapping can also be reduced. However, in the case of an exciplex host with TADF characteristics, the dual exciton recycling action of TADF and exciplex can be observed. So, the accumulation can be reduced by accelerating the exciton distribution and resulting in a longer operational device lifetime. Adachi et al. reported based on this concept and showed that blue OLEDs attained a long lifetime (Nguyen et al., 2020). The TADF-type exciplex host materials showed longer lifespans than conventional and D-A type acceptors. In the case of the host in the device, it offers an overwhelming lifespan improvement effect. When an N-type host (based on the triazine moiety) with TADF characteristics is applied, the exciton transfers back from the first exciplex so that it can be recycled again through the RISC process and effectively distribute exciton (Figure 7). The OLED performance of devices with 50 wt%-Tris-PCz: 50 wt%-acceptor as the emitter is summarized in Table 2. Among the fabricated devices, the exciplex-based OLEDs manifested high EQE values than the control device, indicating the utilization of the potential of TADF exciplex. Furthermore, based on the emission spectra of all devices, changing from the conventional acceptor molecules to TADF-type acceptors only slightly affected both recombination and emissive sites. Among all the devices, the LT_50_ of nearly 300 and 350 h was achieved in the OLEDs based on BCz-TRZ and 3Cz-TRZ, respectively, emphasizing the superiority of the exciplex TADF-type acceptor.

**TABLE 2:** OLED performances for devices with 50 wt%-Tris-PCz (P-type):50 wt%-acceptor (N-type) as the emitter.

P-type (50%)	N-type (50%)	TADF	EQE_500/1000_ (nits)	CIE (x,y))	LT_50 (@ 1000 nits)_ (hrs)
Tris-PCz	3Cz-TRZ	O	8.9/8.4%	(0.26, 0.53)	337
	BCz0TRZ	O	11.9/11.3%	(0.26, 0.50)	292
	Cz-TRZ	X	9.5/9.1%	(0.29, 0.55)	123
	T2T	X	11.5/11.1%	(0.28, 0.54)	66

Among the selected cores, the TADF-type acceptor and bipolar acceptor contain electron-donating groups, which can contribute to the unexpected injection of holes. Subsequently, the enhancement of the operational device lifetime can be attained. Furthermore, such TADF exciplex-type-based OLEDs showed better lifetime, indicating their efficient TADF mechanism. On the other hand, the Cz-TRZ-based OLEDs showed a poor lifetime due to the inefficient RISC process. In contrast, the 3Cz-TRZ showed a longer lifetime, indicating efficient triplet excitons are transferred in exciplex-based OLEDs. Furthermore, the lifetime is fairly better when 1 wt% of conventional MR-type emitter ν-DABNA has been applied to the Tris-PCz: 3CzTRZ combination. However, due to the lower triplet energy and more extended wavelength emission, the devices have poor color coordinates (CIE y = 0.36), which deviates from NTSE and BT2020 standards. However, this exciplex-based host system manifested improved lifetime values compared to a normal host. Moreover, the TADF-type acceptors showed excellent stability due to their efficient RISC process. However, utilizing these acceptors to stable blue OLEDs with support of the exciplex concept, where the broad green and pure blue dopant is utilized, can pave the way for stable HF devices.

### 2.4 Suppression of Dexter energy transfer–shielding approach

Precise control of energy transfer pathways in molecular assemblies is essential in OLEDs, especially in HF devices. DET is the non-radiative electron exchange process from donor to acceptor moieties (Dexter, 2004). In the HF system, the energy loss mechanism occurs through DET from the triplet state of TADF to that of the final emitter, which demands a short distance range for achieving the molecular orbital overlapping (Dexter, 2004; Bai et al., 2020). Figure 2 illustrates the DET mechanism in the HF system, unlike FRET, in which the energy transfer process requires the overlaps of the absorption of energy acceptor with the emission of the energy donor. DET is an electron exchange process that requires the overlapping of frontier molecular orbitals.

**FIGURE 8:**
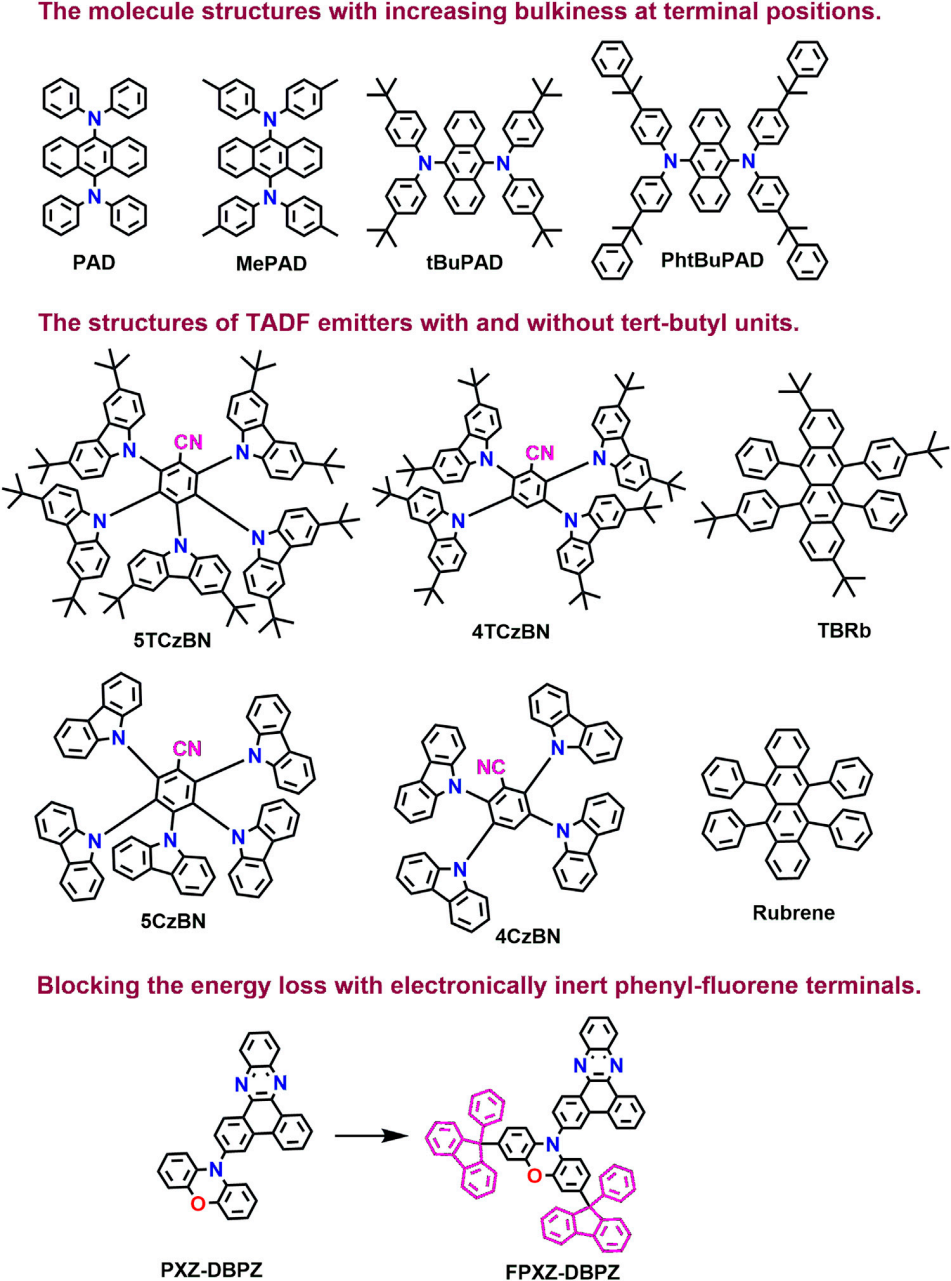
Molecular structures discussed based on DET suppression approach.

Several strategies were adopted to alleviate this DET process without harming FRET efficiency to achieve high efficiency by inhibiting the exciton loss, mainly via DET from T_1_ of the TADF emitter to that of the final emitter (Jeon et al., 2023). Furthermore, the following equation illustrates the rate of DET, which decreases as exp (−2R_DA_/L), where R_DA_ is the donor–acceptor separation relative to their van der Waals radii, L (Wei et al., 2020),

$$k_{\mathrm{DET}}=KJe^{-2R_{DA}/L}$$

where *K*_*DET*_ represents the rate of DET, *k* is related to the specific orbital interactions, and J is the normalized spectral overlapping integral. Duan et al. successfully reduced the DET process by employing bulky substituents such as methyl, tert-butyl, and butyl-phenyl at the terminal edges of N^9^, N^9^, N^10^, N^10^-tetraphenylanthracene-9,10-diamine (PAD). Figure 8 illustrates the series of structures based on PAD realizing the efficiency increase as the inert group’s bulkiness increases (Zhang et al., 2018). This is one of the strategies which efficiently reduces the DET by increasing the intermolecular distance. Furthermore, as bulkiness increases with the inert units from methyl to tert-butyl and butyl-phenyl, they produce a large steric effect. Thus, the inert units not only increase the intermolecular distance but also reduce the orbital overlap of neighboring molecules and, thus, can sterically shield the electronically active cores of emitters. The same group in 2020 realized the high efficiencies, balanced white spectra, and extended lifetimes by modulating the Förster and Dexter interactions in SEL-TSF-WOLEDs (Wei et al., 2020).

As shown in Figure 8, in the molecular structure without tertiary butyl groups, such as 5CzBN, 4CzBN, and Rubrene, the spin-density distributions of triplets and frontier molecule orbitals are distributed all over the whole molecules. At the same time, in the molecular structure with the inert tertiary butyl group at the terminal position of carbazole, such as 5TCzBN, 4TCzBN, and TBRb, distributions of triplets as well as frontier molecule orbitals are mainly located at the core and not extended to the tertiary butyl group at the terminal position of carbazole, suggesting that those electronically inert peripheral units can sterically shield the electronically active chromophores. Furthermore, Su et al. introduced an electronically inert phenyl-fluorene terminal to the PXZ-DBPZ as a TADF sensitizer to block the energy loss through DET (Xie et al., 2022). This blocking effect was investigated by theoretical calculation, which suggests that due to the sp3-hybridized carbon atoms in the phenyl-fluorene units, it does not participate in conjugation and hence does not share the HOMO with the phenoxazine moiety (Figure 8).

Another approach is to up-convert the majority of triplet excitons to singlet excitons by reducing the orbital overlap rather than the transfer to the DET channel. However, to achieve this, the rate constant of RISC (*k*_RISC_) should be high for the TADF sensitizers. Lee et al. proposed the strategy of triplet up-conversion by replacing the donor moiety of 2,3,5,6-tetra(9H-carbazol-9-yl)terephthalonitrile (4CzTPN) with 5H-benzo [4,5]thieno [3,2-c]carbazole (BTCz) (Shin et al., 2022). The BTCz group strengthens the charge transfer characteristic and induces a high RISC rate. Howard et al. demonstrated a shielding effect by substituting the multi-donors in the TADF sensitizer 4CzIPN (Jakoby et al., 2021). The HOMO and LUMO were well-separated by donor–accepter moieties restricting the LUMO–LUMO overlap, resulting in the suppression of triplet transfer. In addition, the separation of HOMO–LUMO by resonance effects suppresses the triplet transfer by dispersion in the host matrix. This strategy is advantageous to inhibit the triplet exciton transfer. Duan et al. reported the sterically hindered BODIPY-type emitter (tPhBODIPY) with a bulk substituent on the meso-position to suppress DET, exhibiting a high photo-luminance quantum yield of 98% and a small FWHM of 28 nm (Song et al., 2020). BODIPY-type emitters are known for their high molar absorption coefficient and sharp emission with narrow FWHM. However, the deep LUMO of tPhBODIPY tends to trap electrons in TADF-sensitized OLEDs. Thus, to reduce the DET process, the TADF architecture with blocking bulky terminal groups, lowering doping concentration and fast *k*_RISC_, and shielded LUMO has the advantage of achieving an efficient HF system.

### 2.5 Molecular orientation of the final dopant

Normally, the EQE can be attained by using the following equation:

$$\eta_{EQE}=\gamma \times \eta_{S/T}\times q_{eff}\times \eta_{out}$$

where γ is the charge balance factor, η_S/T_ is the singlet–triplet factor, q_eff_ is effective quantum yield, and η_out_ is the out-coupling efficiency of the emitter. The η_S/T_ is based on the spin-statistics like η_S/T_ = 0.25 for fluorescent emitters and η_S/T_ = 1 for phosphorescent and TADF emitters. The outcoupling depends on the Purcell factor and emitting dipole orientation (EDO) ratio. The EDO can be represented as the fraction of the horizontal emitting dipole moment, Θ ≡ (*p*_∥_/*p*_∥_ + *p*_⊥_), where *p*_∥_ and *p*_⊥_ represent the horizontal and vertical components of the emitting dipole, respectively. Usually, when light from emitters is arranged horizontally, the surface plasmon loss will be reduced, and the outcoupling efficiency will increase (Kim et al., 2022). So, the higher the degree of molecular orientation of the final emitting dopant, the higher the light extraction efficiency, and the higher EQE can be attained. Furthermore, the EQEs can be enhanced using low refractive index materials, as they support increased outcoupling efficiency of over 60%. These indicate that the Θ can be the most important parameter for efficient OLEDs (Kim and Kim, 2018; Tenopala-Carmona et al., 2021).

For example, recently, many researchers focused on MR-TADF-type materials for OLEDs as they manifested highly recommendable BT2020 standards (Ha et al., 2021; Kim and Yasuda, 2022; Sang Min Cho, 2022). Among the developed MR-TADF materials, the ν-DABNA stood as the benchmark for blue OLEDs, as it has a highly narrow spectrum with high PLQY. By utilizing the ν-DABNA, Adachi et al. developed highly efficient, stable blue OLEDs (Chan et al., 2021). In this work, the HDT-1 was selected as the assistant TADF sensitizer and ν-DABNA as the final emitting dopant. The fabricated device with the emitting layer architecture of 0.5 wt% ν-DABNA: 20 wt% HDT-1: mCBP manifested a high EQE of 27% with a remaining high EQE of 20% even at 1000 nits. Such EQE is higher than that of the device without HDT-1. These EQEs are attributed to the molecular horizontal dipole orientation of the final emitting ν-DABNA in the doped host film.

**FIGURE 9:**
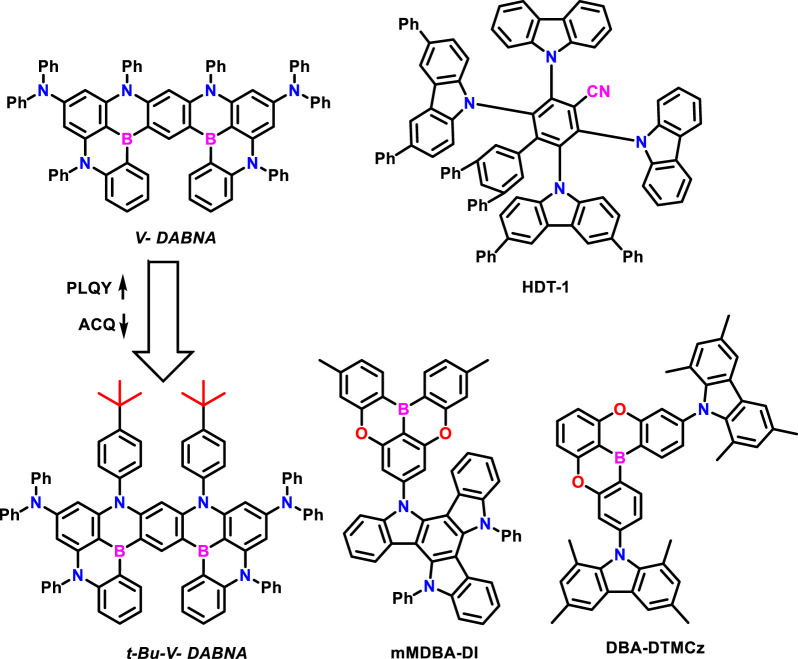
Molecular structures discussed in the molecular orientation part.

Later on, Kwon et al. developed two efficient pure blue TADF assistant dopants for efficient FRET rate to the final dopant (Naveen et al., 2022a). The final dopant was utilized as t-Bu-ν-DABNA, which is tert-butyl-substituted ν-DABNA. The tert-butyl groups in t-Bu-ν-DABNA can enhance the PLQY and reduce the aggregation-caused quenching. Subsequently, the horizontal dipole orientation also improves (Figure 9). Among the developed TADF sensitizers, the mMDBA-DI manifested deep blue color with a high horizontal dipole orientation ratio of 0.82 and PLQY of 97.8%. On the other hand, the t-Bu-ν-DABNA showed PLQY of 91.9%, which is 5.9% lower than that of mMDBA-DI. However, the t-Bu-ν-DABNA showed a higher horizontal dipole orientation ratio of 0.92, which is 0.10 higher than that of mMDBA-DI. So, the differences in device efficiencies entirely depend on the horizontal dipole orientation values. Subsequently, the mMDBA-DI HF device manifested a high EQE of 39.1% with a narrow FWHM of 19 nm in the pure blue region (CIE y = 0.15). Such high EQE is mainly attributed to high horizontal dipole orientation and good TADF characteristics of the final dopant, t-Bu-ν-DABNA. To extend this work, the same group recently reported two efficient TADF sensitizers based on the quadrupolar D-A-D type, namely, DBA-DmICz and DBA-DTMCz. Among the developed sensitizers, DBA-DTMCz with ν-DABNA manifested tremendous EQEmax of 43.9% in the blue region, which is recorded as high EQE among the reported blue devices. Such great EQE was achieved due to the increased horizontal dipole orientation ratio of DBA-DTMCz (Θ = 0.86) (Lee H. et al., 2023).

## 3 Conclusion and outlook

In summary, the rapid development of the HF-OLEDs has resulted in a new dimension of display features by building important information into different device design concepts, exciting and unique electroluminescent behaviors, and, importantly, providing a mechanistic understanding of the pathways of energy transfer processes. This review highlights the relevant factors for building highly efficient and stable HF-OLEDs. The factors are focused on spectral overlapping, energy transfer mechanisms, TSH requirements, electroluminescence study based on the exciplex and polarity system, shielding effect, DET suppression, and FD orientation by considering the device analysis and future developments. Currently, the EQE of the OLEDs was around 40% in wide color gamut regions with the support of the HF system. However, even though high efficiency with proper CIE coordinates is reached, the lifetime still needs improvement, especially in blue HF-OLEDs.

So in the coming days, utilizing the narrowband emitter such as the MR dopant (for the final dopant) and highly efficient TADF (as the sensitizer), which has a high RISC rate (in order of 10^7^ to 10^8^ s^-1^), with more developments in device physics can pave the way for long lifetime HF-OLEDs. The material designs should be focused on stability with high TADF performances. On the other hand, the FRET rate should be improved by minimizing the DET rate values. The rapid developments in this upcoming technology generate enthusiasm, motivating many optoelectronic specialized researchers to explore a deeper understanding and find new advancements in this exciting area of research. This review will provide a clear prospect and attract researchers with diverse interests to these luminescent material developments, highly efficient and stable HF-OLED displays, soon.
